# Supplementary material for: Structural and quantitative alterations of gut microbiota in experimental small bowel obstruction
Source: PLoS One. 2021 Aug 4;16(8):e0255651. doi: 10.1371/journal.pone.0255651 (PMC8336877; doi:10.1371/journal.pone.0255651)
Supplement: Attachment — Submitted filename: Response to Reviewers.docx [file pone.0255651.s005.docx]

Dear editorial board of *PLoS ONE*：

Thank you very much for your letter and advice on our manuscript. We have resubmitted new version of manuscript. We have addressed the comments raised by the reviewers, and the amendments are highlighted in red in the revised manuscript. We hope that the revision is acceptable and look forward to hearing from you soon.

Below, please find the comments in black, followed by our responses in red. Exact changes in the manuscript are also presented in red font.

Replies to Reviewer 2

Q: What gross changes happened in these rats - weight, intestinal diameter, food and water intake, etc.?

A: We have detailed the general condition in the sections of results.

Q: The total volume of the content should be given in these rats and considered in the calculations.

A: We have added the total volume of the content into calculation and the results were presented in Fig 10.

Q: With prolonged obstruction, these rats must be getting significantly dehydrated - how is that taken into account in the data interpretation?

A: We have added some discussions at the end of essay.

Sincerely yours,

Xie JL on behalf of the authors.

Corresponding author: Jiliang Xie, MD, Department of Gastrointestinal Surgery, Nankai Hospital, Nankai District, No.6 Changjiang Rd, Tianjin, 300100, China. Phone: 86-022-27435366. E-mail:[xiejiliangnk@163.com](mailto:xiejiliangnk@163.com)
